# Novel carfilzomib-based combinations as potential therapeutic strategies for liposarcomas

**DOI:** 10.1007/s00018-020-03620-w

**Published:** 2020-08-26

**Authors:** Maya Jeitany, Aishvaryaa Prabhu, Pushkar Dakle, Elina Pathak, Vikas Madan, Deepika Kanojia, Vineeth Mukundan, Yan Yi Jiang, Yosef Landesman, Wai Leong Tam, Dennis Kappei, H. Phillip Koeffler

**Affiliations:** 1 Cancer Science Institute of Singapore, National University of Singapore, Singapore, Singapore; 2 School of Biological Sciences, Nanyang Technological University, Singapore, Singapore; 3 Genome Institute of Singapore, Agency for Science, Technology and Research (A*STAR), Singapore, Singapore; 4 Karyopharm Therapeutics, Newton, MA USA; 5 Department of Biochemistry, Yong Loo Lin School of Medicine, National University of Singapore, Singapore, Singapore; 6 Cedars-Sinai Medical Center, Division of Hematology/Oncology, UCLA School of Medicine, Los Angeles, CA USA; 7 Department of Hematology-Oncology, National University Cancer Institute of Singapore (NCIS), National University Hospital, Singapore, Singapore

**Keywords:** Proteasome inhibitors, Liposarcoma, Combinational therapies

## Abstract

Proteasome inhibitors, such as bortezomib and carfilzomib, have shown efficacy in anti-cancer therapy in hematological diseases but not in solid cancers. Here, we found that liposarcomas (LPS) are susceptible to proteasome inhibition, and identified drugs that synergize with carfilzomib, such as selinexor, an inhibitor of XPO1-mediated nuclear export. Through quantitative nuclear protein profiling and phospho-kinase arrays, we identified potential mode of actions of this combination, including interference with ribosome biogenesis and inhibition of pro-survival kinase PRAS40. Furthermore, by assessing global protein levels changes, FADS2, a key enzyme regulating fatty acids synthesis, was found down-regulated after proteasome inhibition. Interestingly, SC26196, an inhibitor of FADS2, synergized with carfilzomib. Finally, to identify further combinational options, we performed high-throughput drug screening and uncovered novel drug interactions with carfilzomib. For instance, cyclosporin A, a known immunosuppressive agent, enhanced carfilzomib’s efficacy in vitro and in vivo. Altogether, these results demonstrate that carfilzomib and its combinations could be repurposed for LPS clinical management.

**Electronic supplementary material:**

The online version of this article (10.1007/s00018-020-03620-w) contains supplementary material, which is available to authorized users.

## Introduction

The ubiquitin-proteasome system (UPS) is a component that precisely controls the turnover of the majority of proteins [1], making it a critical regulator of numerous cellular pathways. Proper degradation of either misfolded or damaged proteins requires the concerted action of ubiquitin ligases and deubiquitinating enzymes, as well as the proteasome, a multi-subunit complex with proteolytic function. Several pharmacological agents have been developed to target the UPS, including bortezomib and carfilzomib, which inhibit the 20S catalytic core of the proteasome. While these two drugs inhibit the same target, they are structurally distinct. Carfilzomib binds irreversibly to the proteasome, thus sustaining a prolonged inhibition [2]. In addition, carfilzomib shows less peripheral neuropathy in patients [3].

Most cells express the constitutive proteasome, while hematopoietic cells express the immunoproteasome. The active subunits of constitutive proteasomes are β5c (PSMB5), β1c (PSMB6) and β2c (PSMB7) while immunoproteasomes contain instead β5i (PSMB8), β1i (PSMB9) and β2i (PSMB10) subunits [4]. Carfilzomib and bortezomib can target both types of proteasomes by inhibiting the β5c and β5i subunits [5].

Proteasome inhibitors demonstrated to be highly efficient in a number of hematologic malignancies. For instance, bortezomib and carfilzomib are used as one of the standard treatments for multiple myeloma (MM) and relapsed/refractory MM, respectively [6, 7]. However, the efficacy of these drugs as single agents in solid cancers is still debated [8].

Soft tissue sarcomas (STS) are the most frequent type of sarcomas. Liposarcomas (LPS), arising from connective tissues, are the most frequent subtype of STS [9] and can be classified into well-differentiated LPS, dedifferentiated LPS, myxoid/round cell LPS and pleomorphic LPS [10]. While localized tumors can be treated by surgical resection, metastatic or unresectable tumors poorly respond to chemotherapy [11]. Therefore, new therapeutic strategies effective for all subtypes are needed.

In this study, we assess the use of carfilzomib in LPS, as a mono-therapy or in combination with other compounds. We show that carfilzomib alone is sufficient to reduce the viability of LPS cells and the tumor burden of LPS xenograft model. To potentiate carfilzomib’s efficacy, we investigated the use of an inhibitor of XPO1-mediated nuclear export, selinexor, recently described to enhance carfilzomib’s efficacy [12]. To identify further effective drug combinations, we comprehensively profiled changes in the proteomic landscape after proteasome inhibition. Based on these changes, we demonstrate that combining carfilzomib with an inhibitor of FADS2 synergistically reduces the viability of LPS cells. Furthermore, subsequent drug library screening identified novel synergistic carfilzomib-based drug combinations.

## Materials and methods

### Gene expression analyses

Gene expression data was obtained from the CCLE database [13] (v. 20180929) in TPM units. Cell lines with missing ‘Site_Primary’ annotations were excluded from the analysis.

TPM values for GTex [14] and TCGA [15] samples were obtained from UCSC Toil RNAseq Recompute Compendium [16] which reanalyses all samples using a uniform analysis pipeline. Samples of the tissue type ‘Adipose-Subcutaneous’ were included for GTex whereas samples having ‘liposarcoma’ in the histologic_diagnosis were kept for TCGA.

### Cell culture

Liposarcoma cell lines were cultured in RPMI medium supplemented with 10% fetal bovine serum and 1% of Penicillin and Streptomycin. Human adipose-derived stem cells (ASC) were maintained in stromal medium (LaCell). All cell lines were mycoplasma-negative. Source of cell lines, viability and clonogenic assays, generation of shRNA cells, SILAC labeling as well as used drugs are detailed in supplementary data.

### In vivo experiments

Either 0.5, 0.7 or 1 × 10^6^ LPS141 cells were inoculated subcutaneously in the flank of 6–8 weeks old NSG (NOD-SCID gamma) mice. DMSO (vehicle control) or carfilzomib diluted in Citrate buffer (25 mM, pH = 4) + 10% Captisol (MedchemExpress) were administered by intraperitoneal injection on alternate days. For combinational treatments, cyclosporin A diluted in 10% DMSO and olive oil was administered by oral gavage on alternate days. At the end of each experiment, mice were sacrificed; tumors were collected and weighed. All experiments were performed in compliance with ethical regulations of Institutional Animal Care and Use Committee (IACUC) of National University of Singapore.

### Proteomic studies

For drug combination study, SILAC labeled cells were treated for 24 h with selinexor (60 nM) and carfilzomib (15 nM) and nuclear proteins were extracted using Nuclear and Cytoplasmic Protein Extraction Kit (Chongqing Bioseps). For PI-induced proteome changes studies, labeled cells were treated with carfilzomib (80 nM) or bortezomib (40 nM) and proteins were extracted with RIPA buffer (Thermo Fisher Scientific). Details of MS sample preparation and data acquisition, and western blot analysis are provided in supplementary data.

### Phospho-kinase array

MLS402 cells were treated with either carfilzomib, selinexor or combination of both for 24 h. 600 µg of protein extracts were used to perform kinase phosphorylation profiling using Proteome Profiler Human Phospho-Kinase Array Kit (R&D System, ARY003B) according to the manual’s instructions.

### Drug library screening

Around 750 LPS141 cells were plated in 384-well plates. The following day, 317 drugs from the Selleckchem anti-cancer compound library were added using Agilent Bravo Automated Liquid Handling Platform (Agilent) at a final concentration of 1 µM, in addition to DMSO for 3 plates and carfilzomib at 7.5 nM or 15 nM for 2 plates each. 3 days post-treatment, CellTiter-Glo^®^ reagent (Promega) was added using a MultiFlo Microplate Dispenser (BioTek), and luminescence was measured with an Infinite M1000 Pro Microplate Reader (Tecan).

## Results

### PSMB5 is over-expressed in soft tissue sarcomas and is associated with a poor clinical outcome

**Fig. 1:**
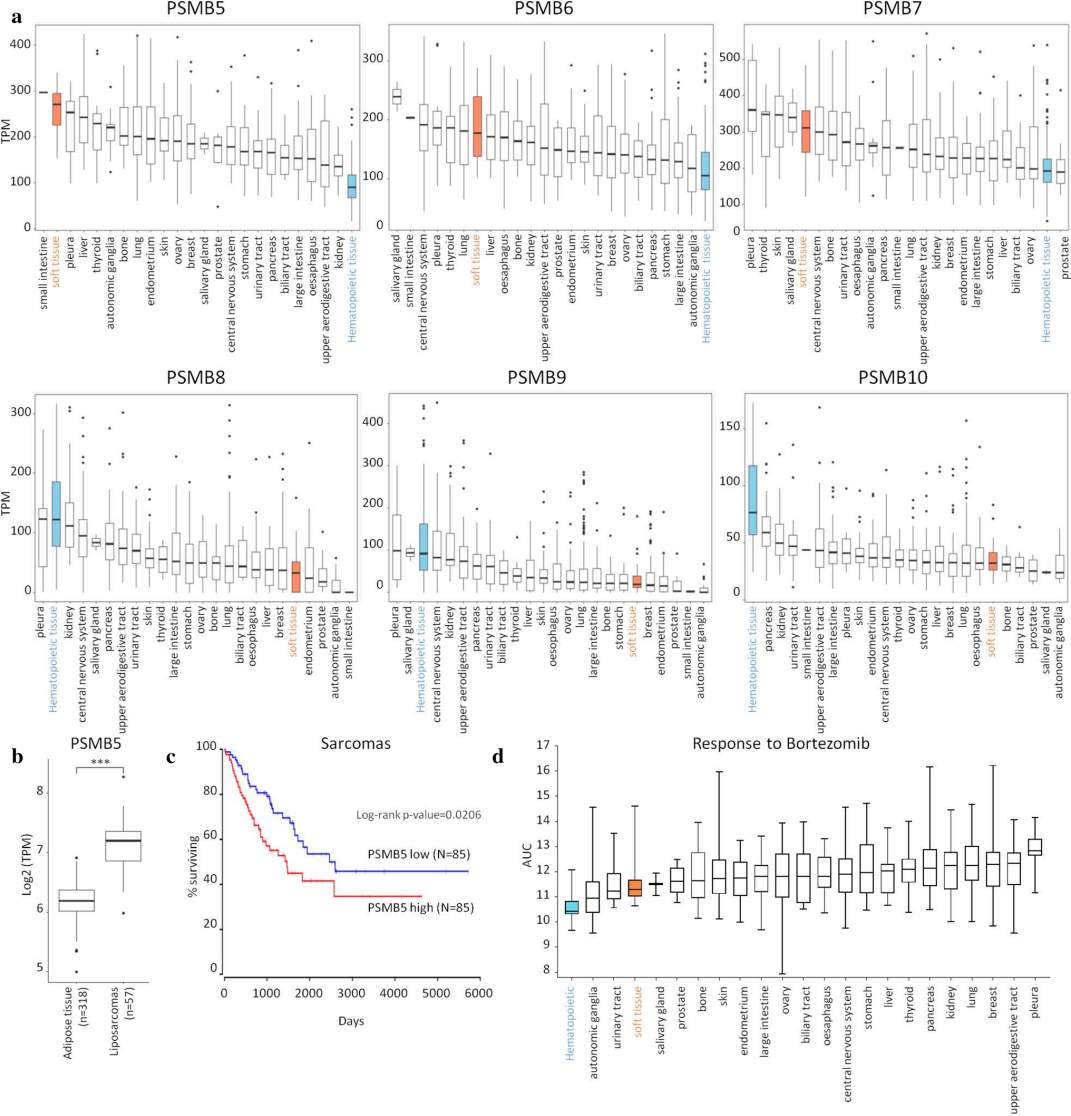
PSMB5 is over-expressed in soft tissue sarcomas and is associated with a poor clinical outcome. **a** Expression of constitutive proteasome subunits (PSMB5, PSMB6, PSMB7) and immunoproteasome subunits (PSMB8, PSMB9, PSMB10) in cell lines encompassing different cancer types. TPM (Transcripts PerKilobase Million) values are plotted with a cut-off of 0.99 percentile. **b** PSMB5 expression in liposarcoma patient samples (TCGA) and normal adipose tissue samples (GTex). N represents the number of samples. Statistical significance was tested using the Wilcoxon rank-sum test. **c** Kaplan–Meier plot showing survival correlation with PSMB5 expression in sarcomas. Values are available in TCGA and were analysed using Oncolnc portal (http://www.oncolnc.org/) [48]. Lower and upper (33:33) percentiles of patients were compared (*N* = 85 for each group). **d** Profiling of response to bortezomib across cell lines belonging to different cancer types. AUC values are downloaded from CTRP (Cancer Therapeutic Response Portal). A lower AUC indicates higher sensitivity

Using CCLE (Cancer Cell Line Encyclopedia) database, we analyzed mRNA levels of the constitutive proteasome and immunoproteasome across available cancer cell lines. In comparison to other cancer types, STS showed high expression of PSMB5, PSMB6 and PSMB7, the subunits of the constitutive proteasome and low levels of PSMB8, PSMB9 and PSMB10, the subunits specific to immunoproteasomes (Fig. 1a).

Moreover, PSMB5, the target of bortezomib and carfilzomib, was found overexpressed in liposarcoma patient samples in comparison to normal adipose tissue (Fig. 1b). Furthermore, survival analysis of sarcomas patients, including liposarcoma patients, revealed that a higher expression of PSMB5 mRNA correlates with a poorer clinical outcome (Fig. 1c).

We then examined the sensitivity of cancer cell lines to bortezomib in the Cancer Therapeutics Response Portal (CTRP; http://www.broadinstitute.org/ctrp). CTRP database calculates AUC values (Areas Under percent viability Curves) which reflects levels of inhibition across all cancer cell lines [17]. Soft tissue cancers exhibited a better response to bortezomib compared to most solid cancers (Fig. 1d). In addition, we recently assessed the effects of 120 drugs on the viability of liposarcoma cell lines covering the major subtypes of LPS [18]. In the presence of bortezomib, reduced viability was seen across all subtypes.

Taken together, these observations indicate a dependency of STS, especially liposarcomas, to the UPS, which prompted us to investigate the efficacy of proteasome inhibitors in LPS.

### Carfilzomib reduces the viability of LPS cells in vitro and in vivo

**Fig. 2:**
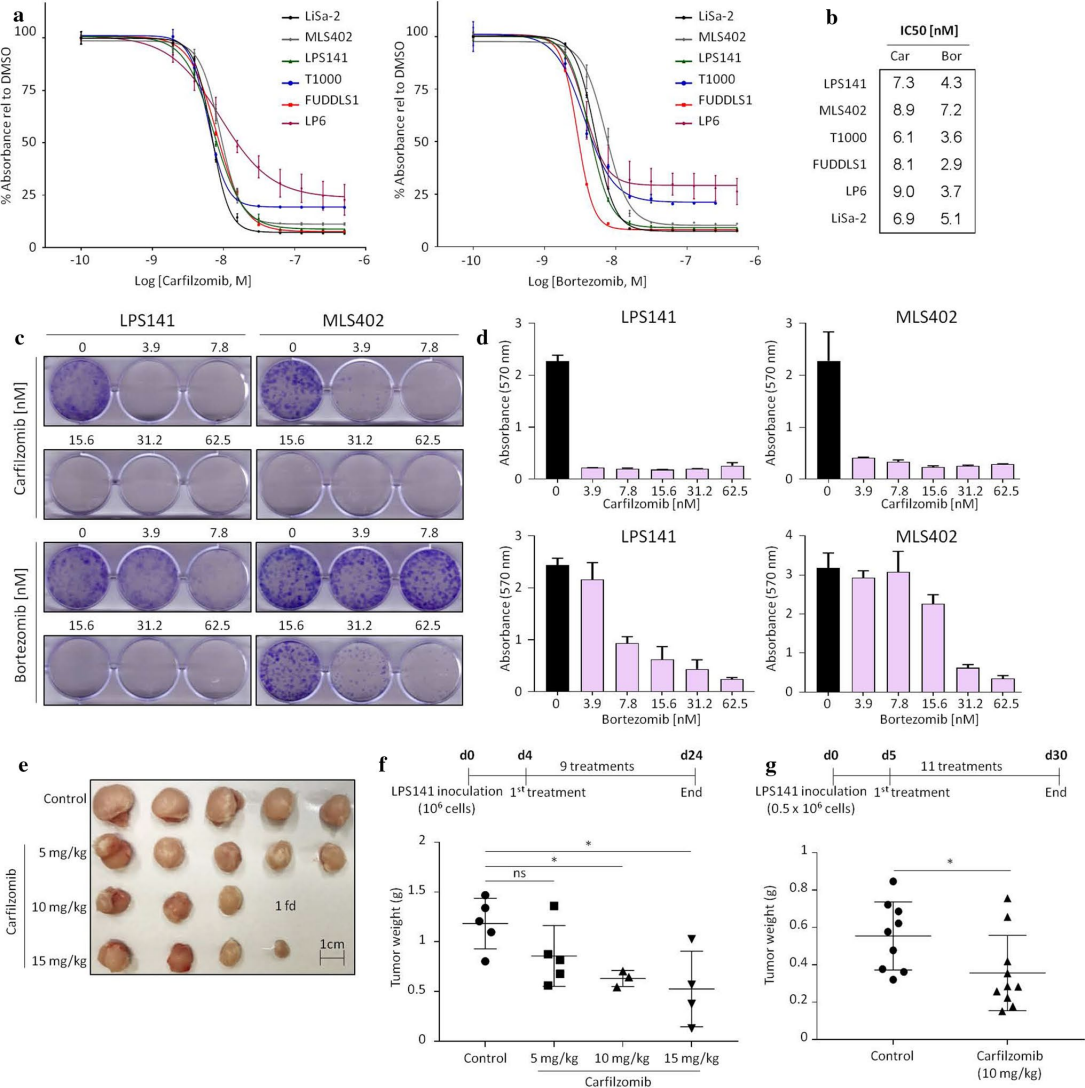
Carfilzomib reduces the viability of liposarcoma cell lines in vitro and in vivo. **a** Dose–response curves of LPS cell lines treated with carfilzomib or bortezomib. Values represent mean ± SD of at least two experiments performed in triplicate. **b** IC50 values from the experiment shown in **a** using GraphPad Prism 7 software. **c** Representative clonogenic assay of LPS cells in the presence of proteasome inhibitors. **d** Absorbance quantification of the clonogenic assays shown in **c**. Values are mean ± SD of duplicate. **e** Tumors excised from mice treated with either vehicle control or carfilzomib. (fd = found dead during the experiment). **f** Experimental design and weight of tumors from the experiment shown in **e** (ns = not significant, *p < 0.05, as determined by two-tailed Mann–Whitney test). **g** Experimental design and weight of tumors from mice treated with vehicle control or carfilzomib (*p < 0.05, as determined by two-tailed Mann–Whitney test)

Sensitivity of different subtypes of LPS cells to bortezomib and carfilzomib was verified in viability assays (Fig. 2a). All cell lines showed reduced viability after 3 days of treatment. Every half-maximal inhibitory concentration (IC50) was in the nanomolar range (< 10 nM) (Fig. 2b), while normal adipose-derived stem cells (ASCs) had an IC50 higher than 250 nM for carfilzomib and higher than 20 nM for bortezomib (Supplementary Figure S1a). The anti-cancer effect of bortezomib and carfilzomib was confirmed by clonogenic assays on LPS141 (dedifferentiated LPS) and MLS402 (myxoid LPS) cells (Fig. 2c, d). Carfilzomib exhibited a better efficacy than bortezomib in reducing colony-forming ability, even at concentrations lower than 3 nM (Supplementary Figure S1b). We, therefore, chose carfilzomib for further testing in vivo. To that end, LPS141 cells were injected sub-cutaneously in immunocompromised mice. 4 to 5 days following injection, either carfilzomib or vehicle control were administered via intraperitoneal route on alternate days (Fig. 2e–g). In a dose-dependent manner, carfilzomib reduced the growth of some LPS141 xenograft tumors (Fig. 2e–g). Mice tolerated the higher concentrations of carfilzomib since a significant change in body weight was not evident (Supplementary Figures S1c, d). These results demonstrate the potential preclinical efficacy of carfilzomib in LPS.

### Selinexor potentiates the effects of carfilzomib in LPS

**Fig. 3:**
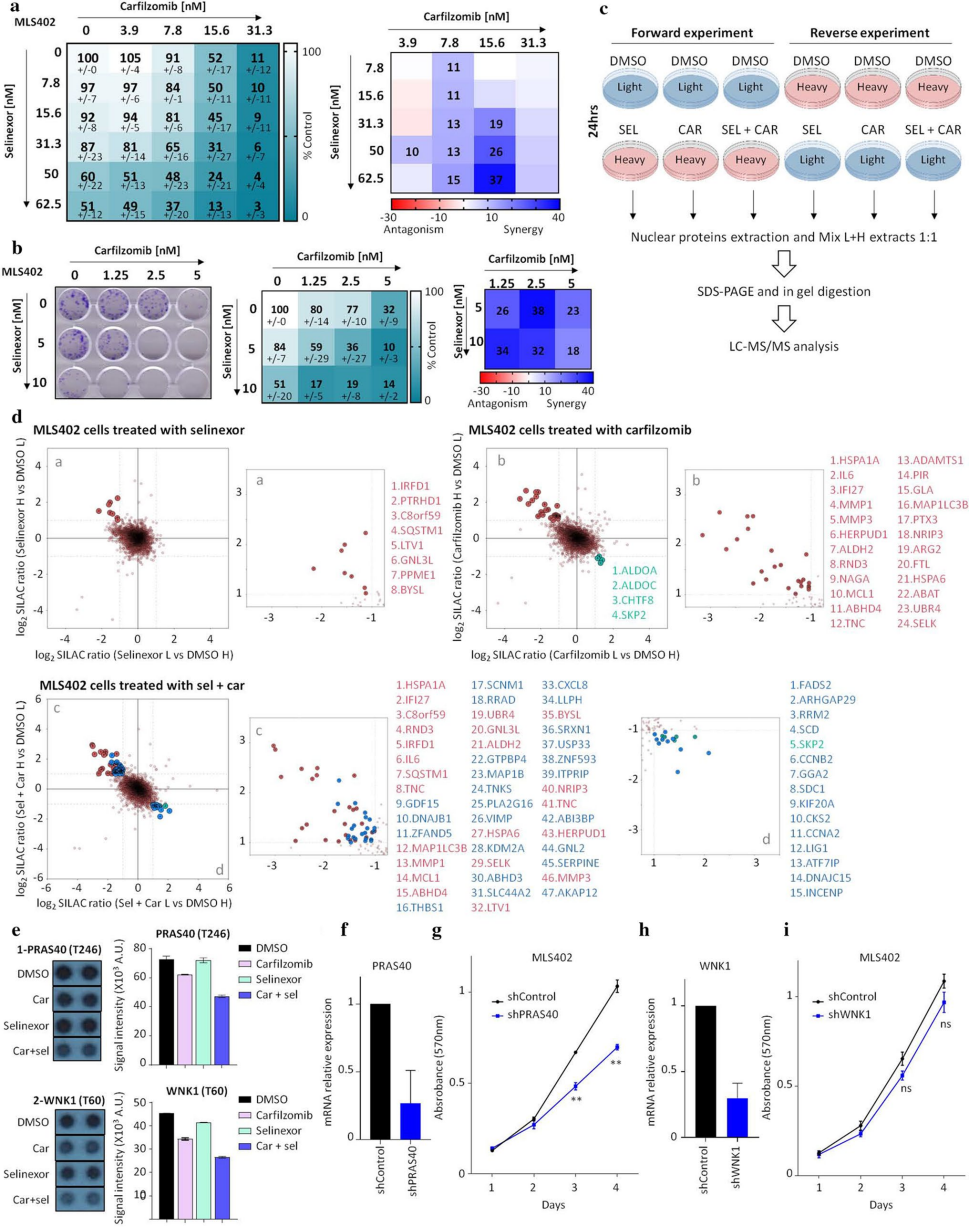
Selinexor potentiates effects of carfilzomib in LPS. **a** Viability assays of MLS402 cells treated with either carfilzomib, selinexor or combinations of both (*n* = 2 experiments performed in duplicate, values indicate mean ± SD). Right panels: heatmaps of HSA (Highest Single Agent) synergy and antagonism scores generated using Combenefit Software [49]. A score < −10 shows antagonism, a score between −10 and 10 shows the additive effect and a score > 10 depicts synergy between the drugs. Only scores ≥ 10 are depicted. **b** Representative Colony formation assay of MLS402 cells treated with combinations of carfilzomib and selinexor. Middle panel represents relative absorbance values ± SD. Right panel depicts the HSA scores. **c** Schematic of SILAC labeling of LPS cells in either ‘heavy’ or ‘light’ medium before drug treatment for 24 h, and subsequent nuclear proteins extraction and processing for LC–MS/MS analysis. **d** Two-dimensional SILAC ratio plots showing quantified proteins by mass spectrometry analysis in MLS402 cells treated with either selinexor (60 nM), carfilzomib (15 nM) or their combination. Proteins in the top left quadrant of each plot are accumulated after drug treatment (log2 fold change > 1), while those on the bottom right are depleted (log2 fold change < −1). Smaller panels are enlarged view of the annotated quadrants (**a**–**d**). Accumulated (in red) or depleted (in green) proteins are annotated. Proteins in blue are those differentially expressed only in the drug combination treatment. **e** Quantification of signal intensities of two phosphorylated kinases (PRAS40 and WNK1) from phospho-kinase arrays of MLS402 treated with either DMSO, carfilzomib (15 nM), selinexor (60 nM) or a combination of both. Left panels are a magnification of highlighted spots in (Supplementary Figure S2C). Right panels show the mean ± SD of signal intensities calculated using Image Studio Lite software. **f**, **h** qRT-PCR analysis of relative expression of PRAS40 or WNK1 transcripts in MLS402 cells transduced with either shControl or shPRAS40 (**f**) or shWNK1 (**h**). Values represent mean ± SEM of three experiments. **g**, **i** Growth curves of MLS402 shControl and shPRAS40 or shWNK1 cells. Values represent mean ± SEM of at least two experiments performed in triplicate. (ns = not significant, **p < 0.01, as determined by two-tailed Mann–Whitney test)

While carfilzomib significantly reduced tumor mass in vivo, tumors were still detected in treated mice. We thus aimed to improve the treatment efficacy through drug combination approaches. Recent studies described a synergy between carfilzomib and selinexor, an inhibitor of XPO1-mediated nuclear export, especially in MM [19–21]. Nair and colleagues also reported that pre-treating sarcoma cells with carfilzomib sensitized the cells to selinexor [12]. In addition, we have previously shown the potency of selinexor against LPS [22]. Therefore, we assessed whether a concomitant treatment of carfilzomib and selinexor can synergistically inhibit LPS viability. Combining both drugs resulted in additive effects at low concentrations and synergy at higher concentrations of selinexor, especially in MLS402 cells (Fig. 3a; Supplementary Figure S2a). A better synergy was noted in colony formation assays where a combination of low doses of each drug was sufficient to inhibit the clonogenic capacity of both cell lines (Fig. 3b; Supplementary Figure S2b).

To delineate cellular changes following both proteasome and XPO1-mediated nuclear export inhibition, we evaluated nuclear protein expression profiles of MLS402 cells by SILAC-based quantitative mass spectrometry, using drug concentrations that exhibited synergy in viability assays. Analysis of accumulated proteins in nuclear fractions has previously shown to be a useful approach for the identification of nuclear export targets of XPO1 [23]. Labeled MLS402 cells (Heavy (H) or Light (L)) were treated for 24 h with either selinexor (60 nM), carfilzomib (15 nM), or both drugs, and nuclear proteins were extracted and subjected to LC–MS/MS analysis (Fig. 3c). For each condition, more than 6900 proteins were identified and an approximate average of 5700 proteins were quantified with H/L SILAC ratios (Supplementary Table 1). Eight proteins accumulated in cells treated with selinexor alone (log2 (fold change) > 1), among which SQSTM1 (a modulator of autophagy of ubiquitinated protein aggregates), LTV1 (a ribosome biogenesis factor), GNL3L (required for ribosomal pre-rRNA processing), and BYSL (essential for the 18S rRNA processing and 40S subunit biogenesis) have been reported to be XPO1 targets [23, 24] (Fig. 3d). Carfilzomib treatment alone induced accumulation of 24 proteins, especially HSPA1A (a proteotoxic stress-induced protein), and depletion of four proteins (Fig. 3d). Differentially expressed proteins (DEPs) identified in drug combination and their corresponding H/L ratios in single treatments are listed in Supplementary Table 1. Some of these DEPs were not measured in single treatments, while other proteins showed a similar expression change tendency in single treatments, and the differential expression became significant and met our threshold criteria only in drug combination (Supplementary Table 1). Interestingly, combining selinexor and carfilzomib resulted in accumulation of other novel proteins, including more proteins required for ribosome biogenesis (GNL2; required for nuclear export and maturation of pre-60S ribosomal subunits, and GTPBP4; potentially involved in the biogenesis of the pre-60S ribosomal subunits [25, 26]), and stress-induced proteins (DNAJB1). These results suggest that this drug combination potentiates each drug’s effects, such as further disrupting ribosome biogenesis and further inducing stress response. Moreover, several proteins were depleted in the combinational treatment, among which FADS2 (Fatty Acid Desaturase 2) was the most depleted.

To identify additional mechanisms contributing to the anti-cancer synergistic effect of selinexor and carfilzomib, we assessed the impact of the drug combination on major signaling pathways. Phospho-kinase arrays of MLS402 cells were profiled after 24 h of treatment with either carfilzomib or selinexor alone, or in combination (Supplementary Figure S2c). Carfilzomib and selinexor alone did not induce significant effects on most kinases, while a combination of both drugs reduced the phosphorylation of two proteins: WNK1 and PRAS40 (Fig. 3e), without affecting the reference spots (Supplementary Figure S2d). WNK1 (WNK lysine deficient protein kinase 1) is a serine/threonine kinase regulating ion transport pathways [27], while PRAS40 (or AKT1S1, Proline-rich Akt1 substrate 1) contributes, among others, to PI3K/Akt and mTOR signaling [28]. Knock-down of PRAS40 (Fig. 3f, g), but not WNK1 (Fig. 3h, i), led to a decrease in MLS402 cell viability. These results highlight an additional mechanistic synergy between carfilzomib and selinexor, potentially leading to the combination’s anti-proliferative effects.

### Proteomic profiles of proteasome inhibition-induced cellular changes in LPS

**Fig. 4:**
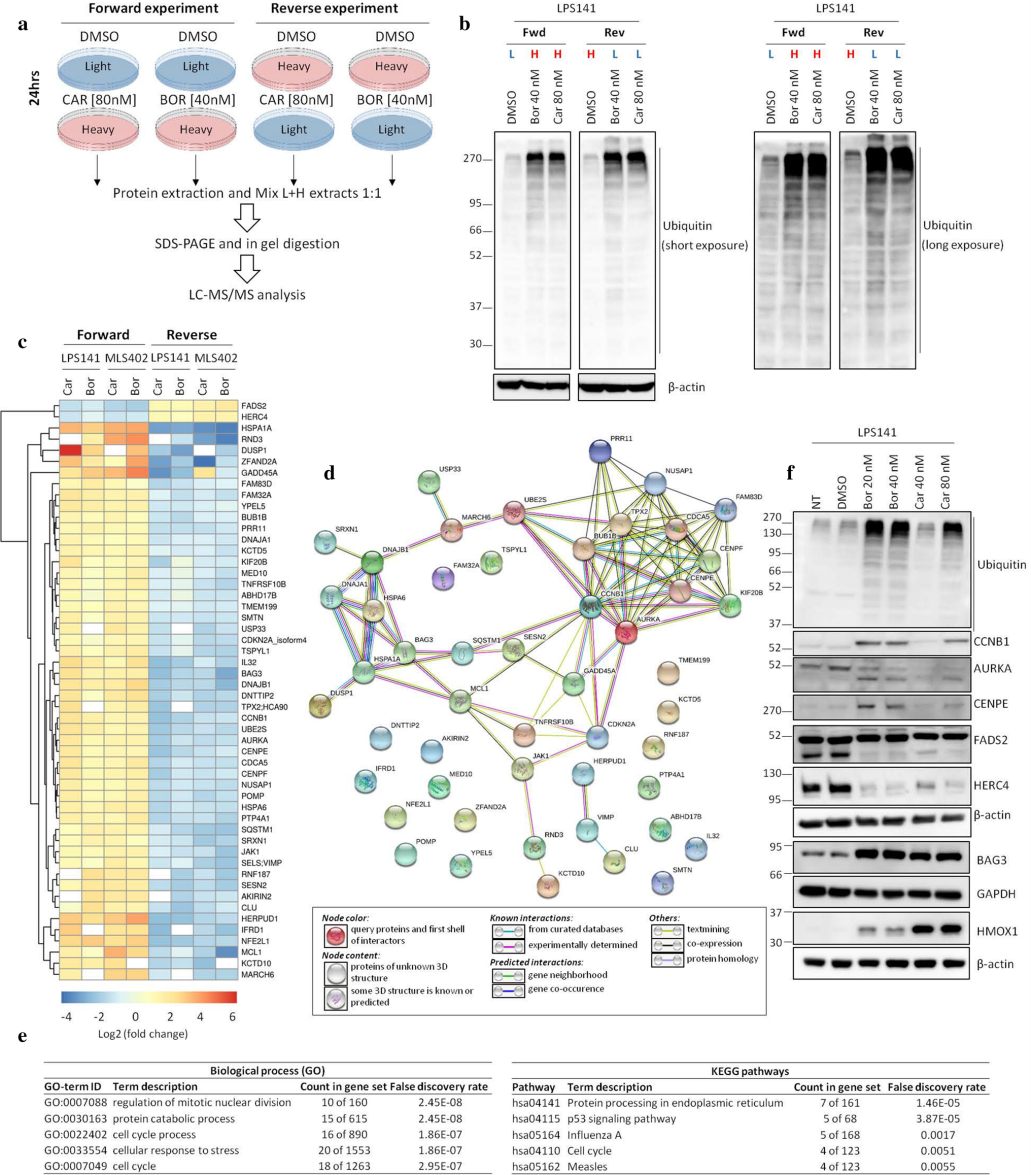
SILAC-based quantitative mass spectrometry analyses of LPS cells treated with proteasome inhibitors. **a** Schematic of SILAC labeling of LPS cells in either ‘heavy’ or ‘light’ medium before drug treatment for 24 h, and subsequent protein extraction for LC–MS/MS analysis. **b** Western blots showing accumulation of ubiquitinated proteins in LPS141 extracts used in proteomic analyses. β-actin serves as a loading control. **c** Heatmap of log2 fold changes (log2fc) of proteins differentially expressed in LPS141 or MLS402 cells treated with either carfilzomib (car) or bortezomib (bor). The ‘forward’ columns represent the log2fc when comparing car- or bor-treated cells grown in ‘heavy’ medium vs. DMSO-treated cells grown in ‘light’ medium. The ‘reverse’ columns represent the log2fc when switching the SILAC labels for DMSO-treated cells vs. car- or bor-treated cells. **d** STRING analysis revealing nodes among the common up-regulated proteins following proteasome inhibition. **e** Biological processes and KEGG pathways enriched among the up-regulated proteins in **d**. **f** Western blots validating a subset of targets identified in **c**. β-actin and GAPDH serve as a loading control

To identify other combinational approaches, global protein expression changes induced by proteasome inhibitors were profiled by quantitative mass spectrometry. Cells cultured in Heavy (H) or Light (L) medium were treated for 24 h with carfilzomib (80 nM) or bortezomib (40 nM) and compared to DMSO-treated cells (Fig. 4a). Treating LPS cells with these inhibitors induced the accumulation of ubiquitinated proteins (Fig. 4b), demonstrating an efficient inhibition of the UPS.

LC–MS/MS proteome analysis identified approximately 7000 proteins and about 5000 proteins were quantified with H/L SILAC ratios in both forward and reverse experiments for each condition (Supplementary Table 2). Fifty proteins accumulated (log2 (fold change) > 1) in both cell lines in at least six out of eight conditions after proteasome inhibition, while only two proteins, FADS2 and HERC4, were commonly down-regulated (log2 (fold change) < −1) (Fig. 4c; Supplementary Figure S3).

A STRING (Search Tool for the Retrieval of Interacting Genes/Proteins) analysis (https://string-db.org) of the up-regulated proteins showed an enrichment of two protein groups; one regulating mitotic nuclear division and cell cycle process (e.g. CENPE, AURKA, TPX2, CCNB1) and another implicated in the cellular response to stress (e.g. BAG3, HSPA6, HSPA1A) (Fig. 4d, e). Either accumulation or reduction of some of the identified proteins were further validated by Western blot (Fig. 4f).

### Additive anti-cancer effect of carfilzomib and HSF1 inhibition

To assess whether changes in protein levels result from transcriptional regulation, mRNA levels of some targets were measured. mRNA expression changes did not always correspond to protein level changes, and even showed an opposite effect in some cases (Supplementary Figure S4a). In agreement with protein quantifications, an induction of mRNAs of cellular stress response genes, such as HSPA6, HMOX1, BAG3, and IRFD1, was observed (Supplementary Figure S4B). Transcription of these genes can be induced by the stress-activated transcription factor HSF1 (Heat Shock Factor protein 1) [29, 30].

We tested whether a chemical inhibitor of HSF1 (KRIBB11) can further sensitize LPS cells to carfilzomib. In both viability (Supplementary Figures S4c-d) and clonogenic assays (Supplementary Figures S4e, f), combining KRIBB11 and carfilzomib showed a mild synergy at some concentrations, while most of the combinations showed only additive effects.

### Carfilzomib and FADS2 inhibitor synergistically reduce LPS viability

Two proteins were down-regulated after proteasome inhibition: FADS2 (Fatty Acid Desaturase 2) and HERC4 (probable E3 ubiquitin-protein ligase HERC4) (Fig. 4c–f). Given their consistent down-regulation, and the fact that FADS2 was found down-regulated after combinational treatment of selinexor and carfilzomib (Fig. 3d), we tested whether FADS2 and HERC4 play a role in carfilzomib’s anti-cancer effects. Due to the absence of HERC4 inhibitors, we down-regulated HERC4 using siRNAs or CRISPR-Cas9 strategy and found that knocking-down HERC4 did not affect the survival of LPS cells nor their response to carfilzomib (data not shown).

**Fig. 5:**
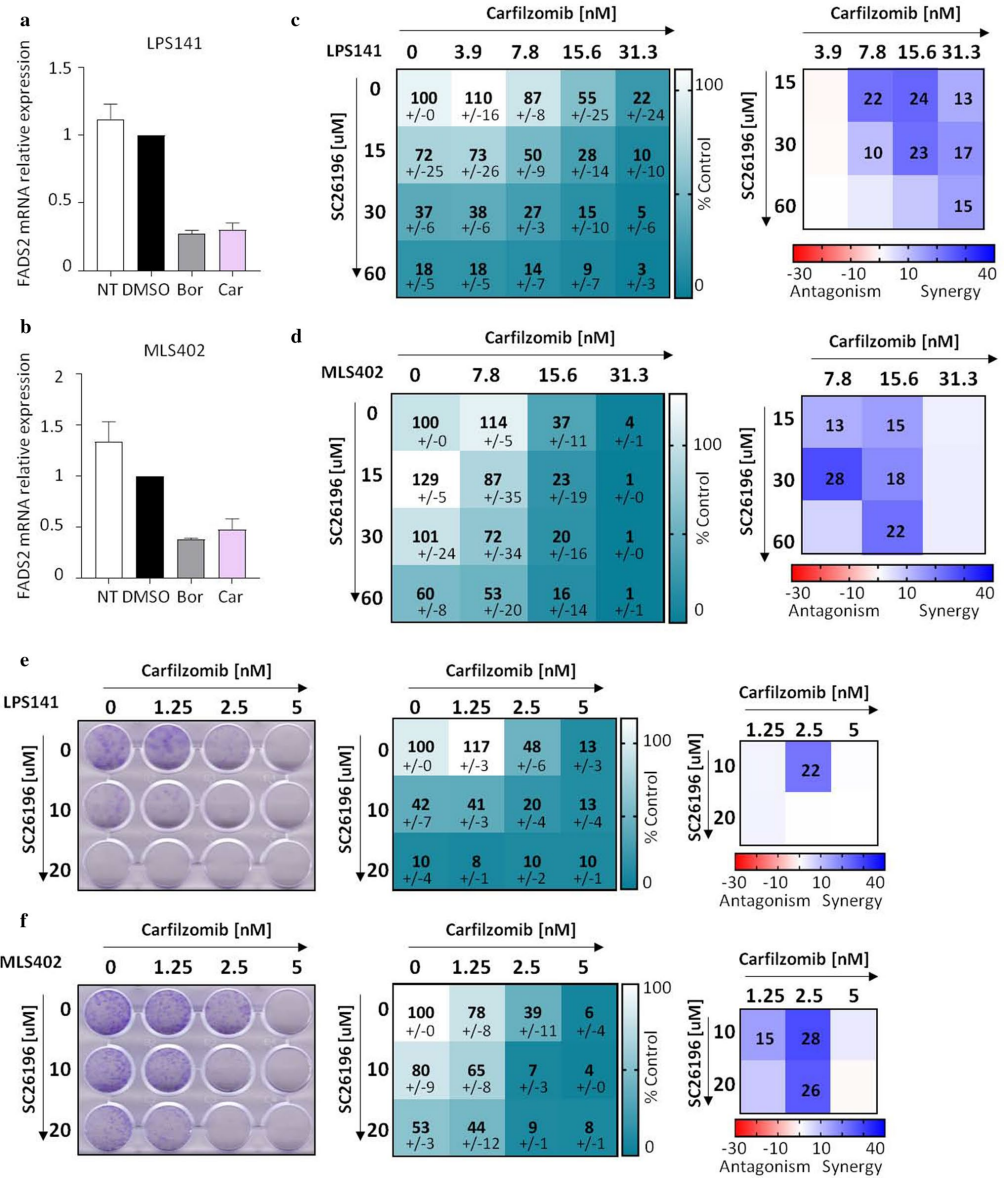
Carfilzomib and FADS2 inhibitor can synergistically reduce LPS viability. **a**, **b** qRT-PCR showing mRNA relative expression of FADS2 in LPS141 (**a**) or MLS402 (**b**) cells non-treated (NT), treated with either DMSO, bortezomib (40 nM) or carfilzomib (80 nM) for 24 h. Values represent mean ± SEM of at least two experiments. **c**, **d** Left panels: heatmaps depict viability in either LPS141 (**c**) or MLS402 (**d**) cell lines after treatment with either SC26196 (FADS2 inhibitor) or carfilzomib or combinations of both. Values represent mean ± SD of percentage of absorbance relative to DMSO of at least two experiments performed in duplicate. Right panel is a heatmap of the HSA scores. **e**, **f** Representative clonogenic assays after treatment of LPS141 (**e**) and MLS402 (**f**) cell lines with combinations of carfilzomib and SC26196. Left panels are representative images of the assays. Middle panels represent relative absorbance values of each condition. Values are mean ± SD. Right panels depict the HSA synergistic scores (> 10)

FADS2 is a key enzyme implicated in the biosynthesis of polyunsaturated fatty acids [31]. FADS2 mRNA transcripts were down-regulated upon proteasome inhibition (Fig. 5a, b). To assess whether FADS2 down-regulation is detrimental for LPS cells, LPS cells were treated with a chemical inhibitor of FADS2 (SC26196) [32]. SC26196 reduced the viability of LPS cells and showed synergy when combined with carfilzomib in viability (Fig. 5c, d) and clonogenic assays (Fig. 5e, f). These observations demonstrate that FADS2 inhibition in LPS cells could enhance carfilzomib’s anti-cancer potency.

### Drug library screen identifies novel carfilzomib-based drug combinations for LPS

**Fig. 6:**
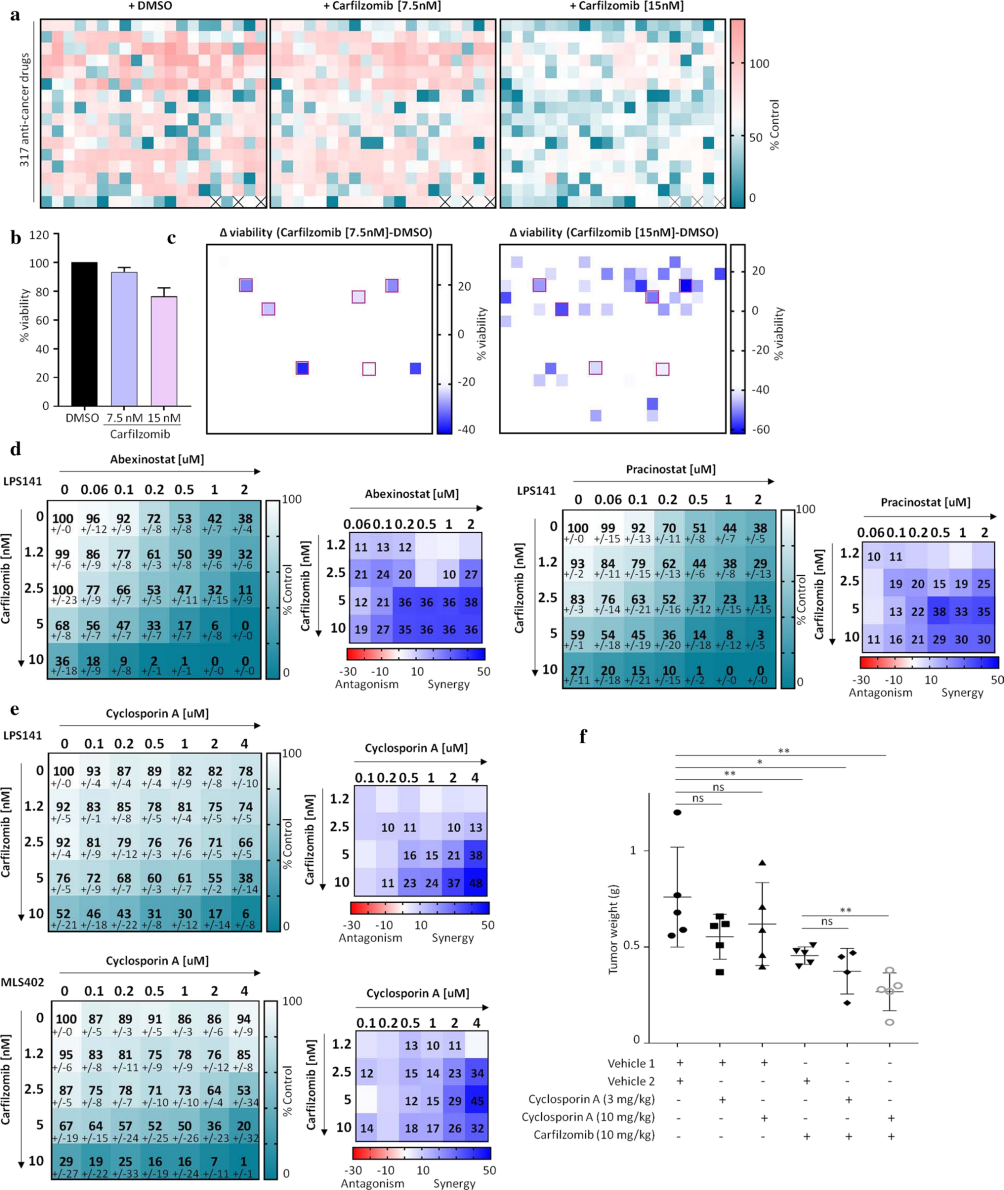
Drug library screen identifies novel carfilzomib-based drug combinations for LPS. **a** Drug library screening of LPS141 cells. Each heatmap represents viability as a percentage relative to control cells, assessed by Cell-titer Glo. Values represent the average of at least two replicates of plates treated in addition with either DMSO, carfilzomib (7.5 nM) or carfilzomib (15 nM). **b** Percentage of the viability of LPS141 cells treated with either 7.5 nM or 15 nM of carfilzomib alone in (6**a**). **c** Differential values of viability between carfilzomib (7.5 or 15 nM) and DMSO-treated cells. Wells highlighted with shades of blue represent a difference of at least either 20% (left panel) or 40% (right panel) of viability. Wells with red borders are drugs identified in both screens. **d**, **e** Viability assays of cells treated with combinations of carfilzomib and either abexinostat or pracinostat (**d**) or cyclosporin A (**e**) (*n* = 2 experiments performed in duplicate; values are mean ± SD). Right panels are heatmaps of the HSA scores. **f** Weight of tumors from mice treated with either vehicle controls, 3 or 10 mg/kg of cyclosporin A, 10 mg/kg of carfilzomib or combinations of both. (*p < 0.05, **p < 0.01, ns = not significant, as determined by two-tailed Mann–Whitney test)

Since no FADS2 inhibitor is clinically approved to date, we searched for FDA (Food and Drug Administration)-approved drugs that could potentiate carfilzomib’s efficiency. Viability of LPS141 cells was assessed in the presence of 317 anti-cancer compounds, combined or not with carfilzomib (7.5 or 15 nM) (Fig. 6a). In this screen, carfilzomib at 7.5 nM alone did not show an effect while at 15 nM reduced viability by around 20% (Fig. 6b). Compared to DMSO-treated plates, drugs that induced either > 20% or > 40% of decreased viability in the carfilzomib [7.5 nM] or [15 nM]-treated plates, respectively, were identified (Fig. 6c; Supplementary Figure S5a). Thirty-seven drugs, of which seven are HDAC inhibitors, showed potential synergy with carfilzomib (Supplementary Figure S5a). Six drugs (cyclosporin A, medroxyprogesterone acetate, abexinostat, aprepitant, GSK1904529A and pracinostat) were identified in both screens. Synergies between carfilzomib and either abexinostat or pracinostat (two HDAC inhibitors) were subsequently validated (Fig. 6d, Supplementary Figure S5b). Likewise, the synergy between carfilzomib and cyclosporin A was confirmed (Fig. 6e). In vivo, cyclosporin A (at 10 mg/kg) significantly reduced tumor burden when combined with carfilzomib, but not when administered alone (Fig. 6f). This combination did not affect the mice body weight (Supplementary Figure S5c), showing that combining carfilzomib and cyclosporin A represents a safe and efficacious potential strategy for LPS treatment.

## Discussion

Although proteasome inhibition (PI) has become a cornerstone in the clinical management of MM, proteasome inhibitors have been disappointing in solid cancers. Here, we show that carfilzomib is potent against liposarcomas, alone or in combinational treatment.

Selinexor, a nuclear transport inhibitor recently approved by the US FDA for MM [33], has shown anti-cancer activity in patients with dedifferentiated LPS in a phase IB clinical study [34]. A recent study showed that selinexor potentiates carfilzomib’s effects in sarcomas [12]. The authors proposed that this is due to NFkB inhibition, an observation reported in other cellular models [19, 21]. XPO1 is responsible for nuclear export of various proteins, including newly assembled 40S and 60S ribosomal subunits [35, 36]. In agreement with that, our nuclear proteome profiling indicated that combining selinexor and carfilzomib interferes with ribosome biogenesis networks. It was recently reported that selinexor synergizes with Bcl-xL inhibitors to induce apoptosis in cancer cells, by impeding rRNA processing and reducing levels of mature rRNAs, necessary for protein translation [37]. A similar concerted action of selinexor and carfilzomib on protein synthesis may be one of the causes leading to reduced LPS cell viability. Additionally, we identified another probable mode of action of this combination: inhibition of PRAS40, a pro-survival protein.

Various events have been previously described as contributors to PI-induced cell death, including induction of endoplasmic reticulum (ER)-stress, induction of apoptosis and/or inhibition of NFkB pathway [38]. We comprehensively delineated protein expression changes induced by PI in LPS cells, identified a recurrent down-regulation of FADS2 following PI, and demonstrated synergy between carfilzomib and an inhibitor of FADS2. FADS2 is a key enzyme of lipid metabolism and fatty acid biogenesis. These pathways are crucial to lipogenic malignancies [39] and were proposed as therapeutic targets in LPS [40]. Given the pro-tumoral roles of perturbed lipid metabolism in cancers [41], a dual inhibition of the proteasome and FADS2 could enhance the potency of proteasome inhibitors in solid cancers. In support of this, pharmacological inhibition of another key enzyme in the fatty acid synthesis pathway, FASN (Fatty Acid Synthase), enhanced the effects of bortezomib in prostate cancer cells [42].

Finally, through drug library screening, we identified more drugs synergizing with carfilzomib, such as cyclosporin A and HDAC inhibitors. Synergy between HDAC inhibitors and proteasome inhibitors has been documented in various types of cancers [43, 44]. Cyclosporin A is an immunosuppressive agent used to prevent organ rejection after transplantation as well as to treat several inflammatory disorders [45]. Clinical doses of cyclosporin A depend on its application. It can be given up to 20 mg/kg per day after organ transplantation; while in other diseases, it is used at lower doses [45]. Studies have investigated the use of low doses of cyclosporin A as anti-cancer therapy, mostly in combination with other drugs, and suggested that cyclosporin A may be valuable in cancer treatment [45–47]. The mechanisms underlying these effects are, however, unclear. We here showed that cyclosporin A potentiated carfilzomib’s capacity to reduce tumor burden. A further optimization of the use of lower doses of cyclosporin A and a deeper understanding of the mode of actions of this combination would be worthwhile, given that cyclosporin A could represent an affordable anti-cancer drug.

Overall, we propose that carfilzomib could be repurposed to treat liposarcoma patients. We show that it can be used in the broad range of LPS subtypes, as a single agent, or in combination with other preclinical, clinical or FDA-approved drugs.

### Electronic supplementary material

Below is the link to the electronic supplementary material.

**Supplementary Table** **1:** List of quantified proteins in the LC–MS/MS nuclear proteome profiling of both forward and reverse experiments of MLS402 cells treated with either selinexor, carfilzomib, or a combination of both. Differentially expressed proteins (DEPs) identified in the combination are also shown in a separate worksheet, with the corresponding H/L ratios in single-drug treatments. NM = not measured. Hits in blue are those meeting the threshold criteria (ratio H/L > 2 and < 0.5) only in the drug combination

**Supplementary Table** **2:** List of quantified proteins in the LC–MS/MS proteome analysis of both forward and reverse experiments of MLS402 and LPS141 cells treated with either carfilzomib or bortezomib.

**Supplementary Figure S1: a)** Viability of Adipose-derived Stem Cells (ASCs) after treatment with either carfilzomib or bortezomib. Values represent mean ± SD of at least triplicate. **b)** Representative clonogenic assay of LPS cells treated with Carfilzomib. **c**, **d)** Bodyweight graphs of mice used in Fig. 1e–g. **Supplementary Figure S2: a)** Viability assays of LPS141 cells treated with either carfilzomib, selinexor or combinations of both (n = 2 experiments performed in duplicate, values are mean ± SD). Right panel is a heatmap of the HSA synergy and antagonism scores.**b)** Colony formation assays of LPS141 cells treated with combinations of carfilzomib and selinexor. Left panel is a representative image of the assay. Middle panel represents relative absorbance values of each condition. Right panel depicts the HSA scores. **c)** Phospho-kinase arrays on MLS402 cells treated with either DMSO, carfilzomib (15 nM), selinexor (60 nM) or a combination of both. Antibodies against phosphorylated forms of 43 kinases and 2 related total proteins are spotted on the membrane in duplicate. A chemiluminescent signal represents phosphorylation of each protein. **d)** Quantification of signal intensities of the reference spots on the phospho-kinome array. **Supplementary Figure S3:** Two-dimensional SILAC ratio plots showing quantified proteins by mass spectrometry analysis in either LPS141 or MLS402 cells treated with either carfilzomib or bortezomib. Proteins in the top left quadrant of each plot are accumulated after proteasome inhibition, while those on the bottom right are down-regulated. Proteins annotated on the right side of each plot are those which accumulated (log2 fold change > 2) after treatment with either carfilzomib or bortezomib.**Supplementary Figure S4: a-b)** qRT-PCR showing relative expression of selected transcripts in either LPS141 non-treated (NT), treated with either DMSO, bortezomib (40 nM) or carfilzomib (80 nM) for 24 h. Values represent mean ± SEM of three experiments. **c-d)** Left panel: heatmap of viability assays of LPS141 (c) and MLS402 (d) cell lines after treatment with increasing concentrations of either KRIBB11 (HSF1 inhibitor), carfilzomib or combinations of both. Values represent mean ± SD of the percentage of proliferation relative to DMSO control of at least two experiments performed in duplicate. Right panel: heat map of HSA synergy and antagonism scores. **e–f)** Colony formation assays after treatment of LPS141 (e) and MLS402 (f) cells with combinations of carfilzomib and KRIBB11. Left panel is a representative image of the assay. Middle panel represents relative absorbance values of each condition. (Representative experiment performed in triplicate; values are mean ± SD). Right panels depict the HSA scores. **Supplementary Figure S5: a)** List of drugs identified in Fig. 5b. The table includes the major targets of each drug. Highlighted drugs are those found in both screens. **b)** Viability assays of MLS402 cells treated with combinations of carfilzomib and either abexinostat or pracinostat (n = 2 experiments performed in duplicate; values are mean ± SD). Lower panels are heatmaps of the HSA synergy and antagonism scores for each heatmap. **c)** Experimental design for the in vivo experiment using LPS141 xenograft models and body weight curves of mice used in Fig. 6f

Supplementary material 4 (DOCX 25 kb)

## Data Availability

The mass spectrometry proteomics data have been deposited to the ProteomeXchange Consortium via the PRIDE repository with the dataset identifiers PXD021001 and PXD020906.
