# Supplementary material for: Novel carfilzomib-based combinations as potential therapeutic strategies for liposarcomas
Source: Cell Mol Life Sci. 2020 Aug 26;78(4):1837–51. doi: 10.1007/s00018-020-03620-w (PMC7904719; doi:10.1007/s00018-020-03620-w)

Supplementary Figure S1:

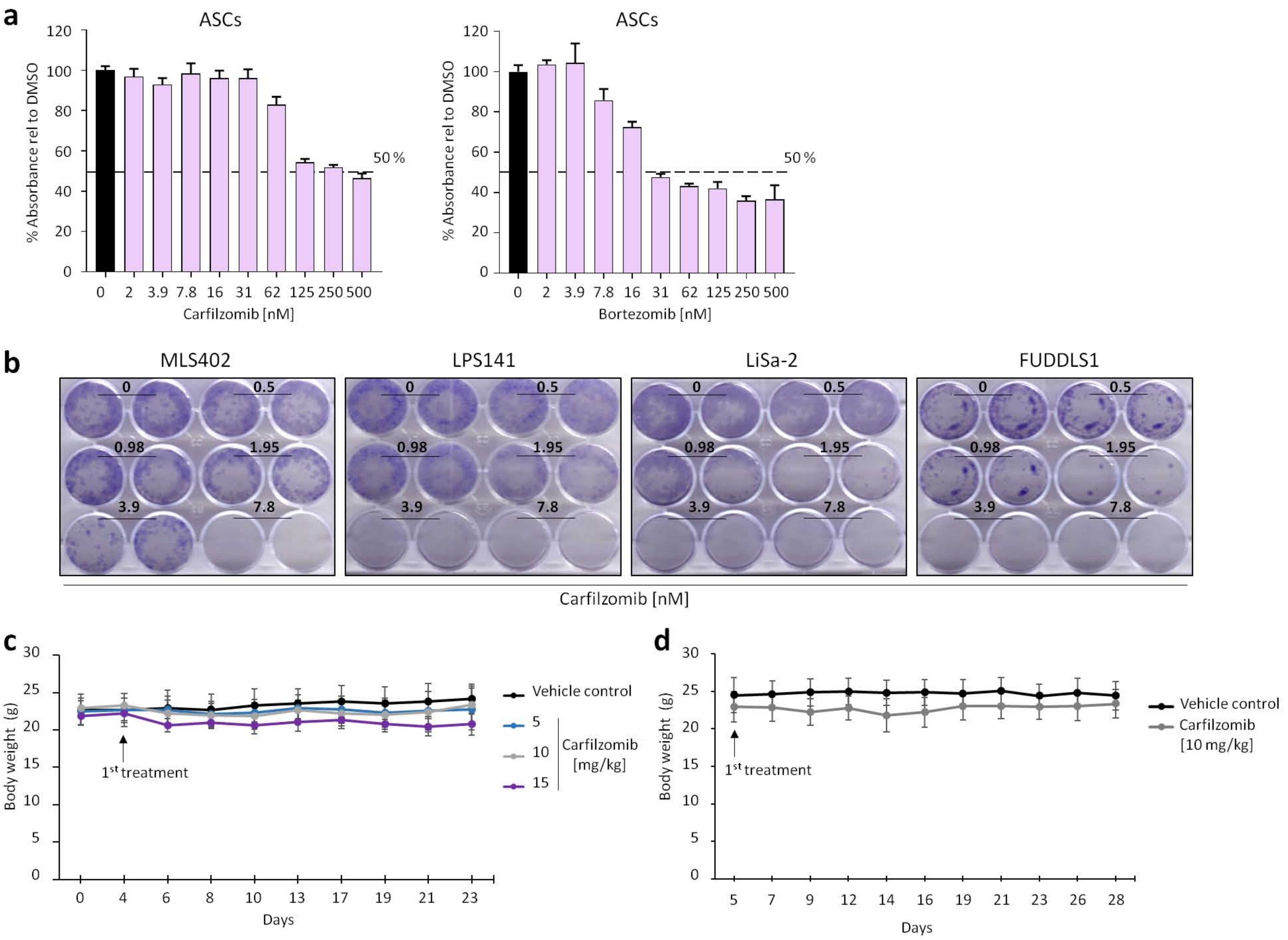

Supplementary Figure S2:

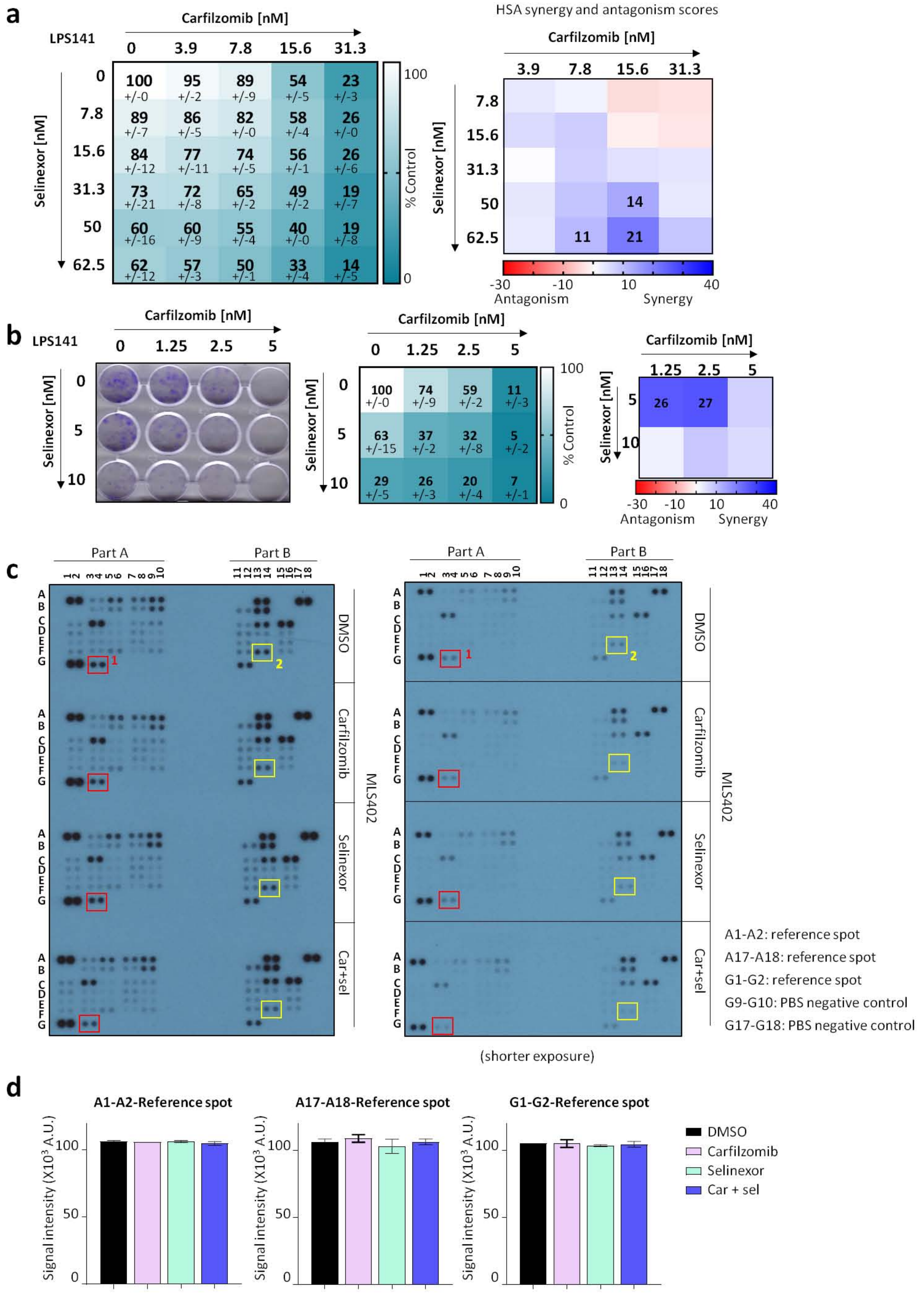

Supplementary Figure S3:

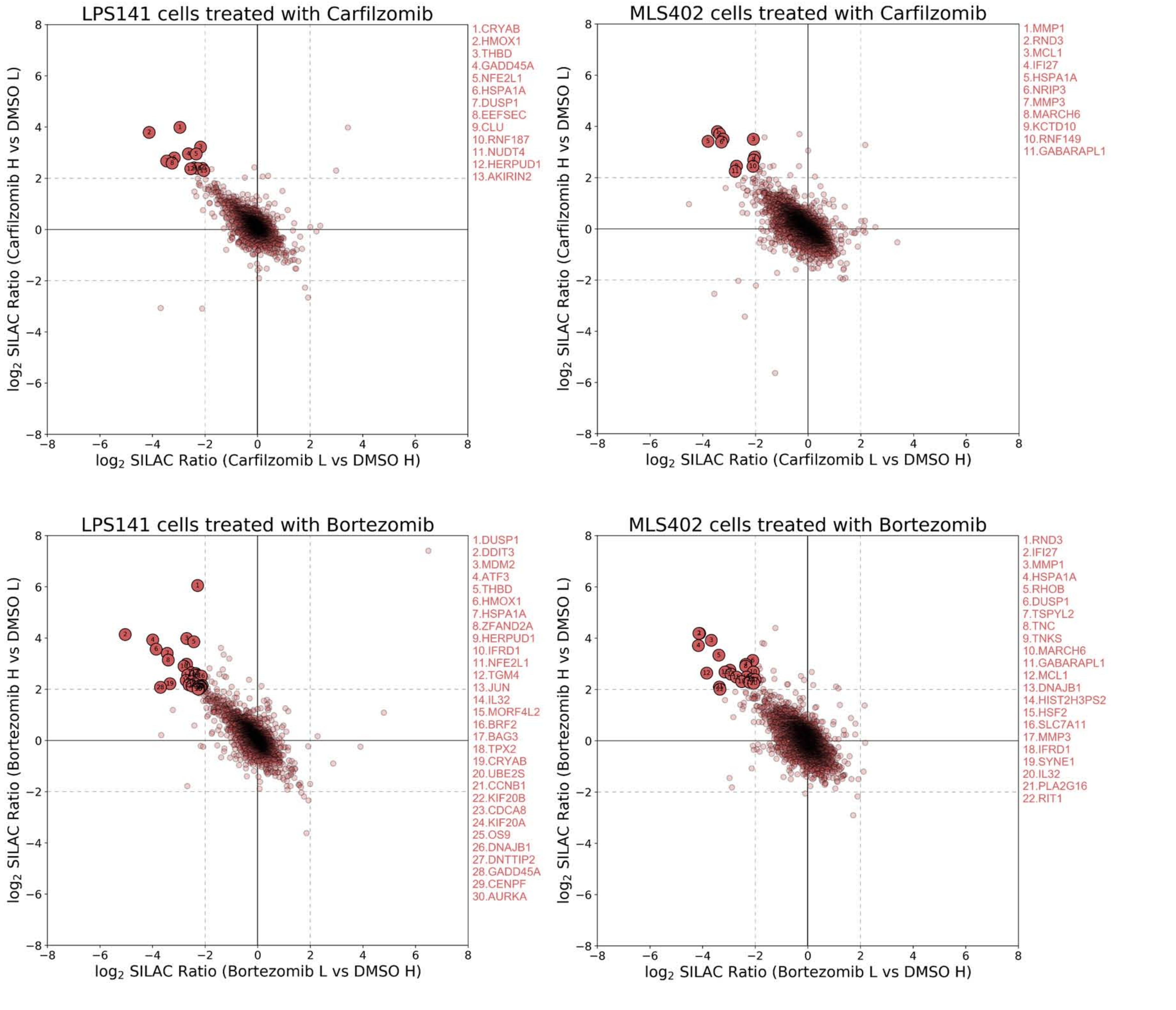

Supplementary Figure S4:

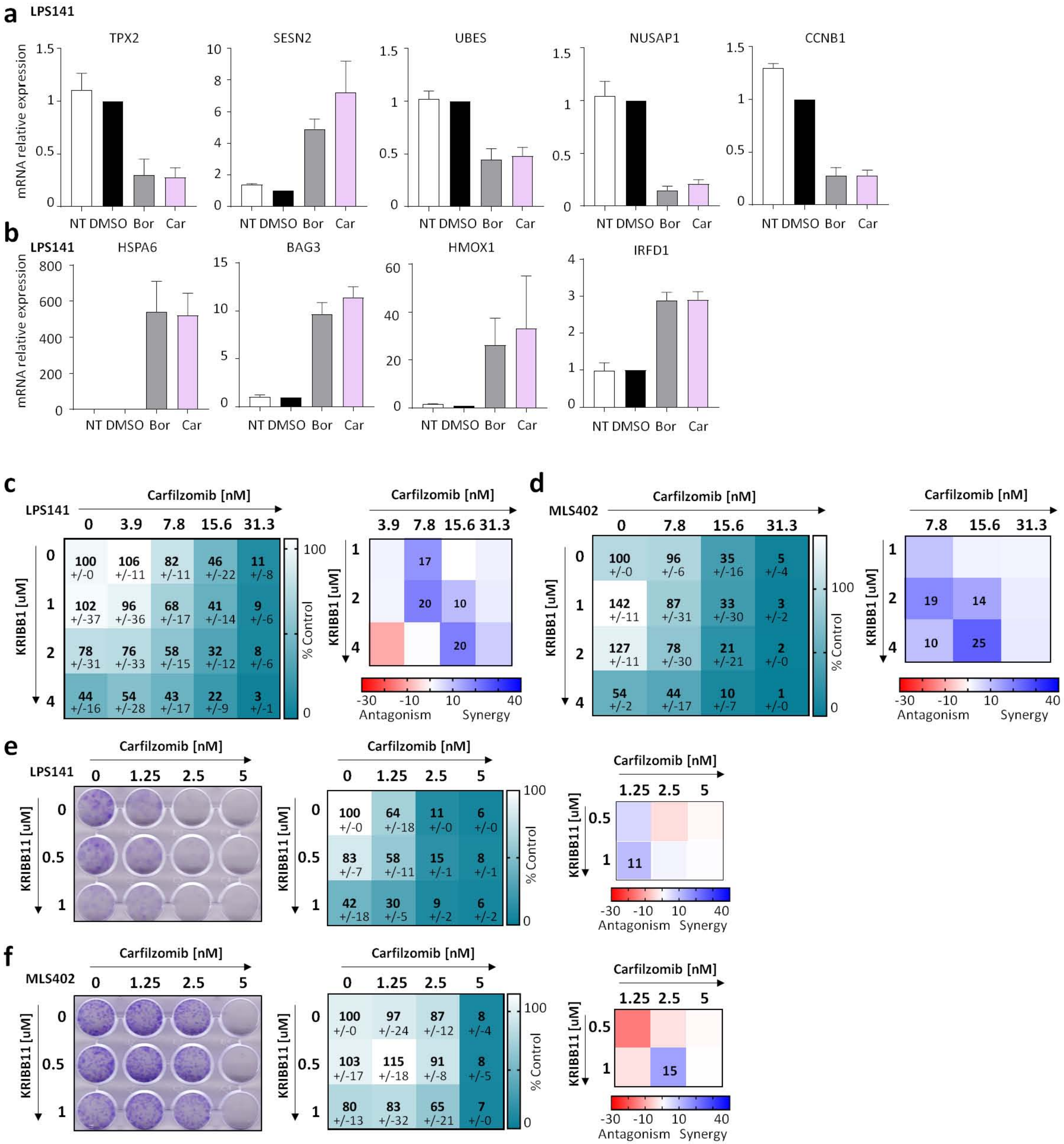

Supplementary Figure S5:

a

Drug name	Major targets
SB 525334	ALK5
Nilotinib (AMN-107)	Abl
Vorinostat (SAHA)	HDAC
Cyclosporin A (Cyclosporine A)	cyclophilin
Rosiglitazone (Avandia)	PPARγ
AZ628	B-Raf, B-RafV600E, and c-Raf-1
CI-1040 (PD184352)	MEK1
Belinostat (PXD101)	HDAC
Medroxyprogesterone acetate	progesterone receptor agonist
AT-406	XIAP, cIAP1, and cIAP2
Telatinib (BAY 57-9352)	VEGFR2, VEGFR3, PDGFα, and c-Kit
Phloretin (Dihydronaringenin)	active transport of glucose into cells by SGLT1 and SGLT2
Pioglitazone (Actos)	PPARγ agonist
PCI-24781 (Abexinostat)	HDAC
Mocetinostat (MGCD0103)	HDAC
Cisplatin	DNA cross-linker
Mifepristone (Mifeprex)	progesterone and glucocorticoid receptors antagonist
Valproic acid sodium salt	HDAC1
Quercetin (Sophoretin)	PI3K
Dalcetrapib (JTT-705)	rhCETP
Gefitinib (Iressa)	EGFR
LY2228820	p38 MAPK α/β
Coenzyme Q10 (CoQ10)	antioxidant agent
Aprepitant (MK-0869)	neurokinin 1 receptor antagonist
Desmethyl Erlotinib (CP-473420)	EGFR
Megestrol Acetate	HegG2
Imatinib Mesylate	c-Kit, Bcr-Abl, and PDGFR
Febuxostat (Uloric)	xanthine oxidase
GSK1904529A	IGF-1R and IR
AR-42 (HDAC-42)	HDAC
Bicalutamide (Casodex)	androgen receptor
Lapatinib Ditosylate (Tykerb)	EGFR and ErbB2
SB939 (Pracinostat)	HDAC
Dapagliflozin	sodium-glucose co-transporter 2 (SGLT2)
Imatinib (Gleevec)	c-Kit, Bcr-Abl, and PDGFR
Fulvestrant (Faslodex)	Estrogen Receptor antagonist
Ostarine (MK-2866)	Androgen Receptor Modulator

b

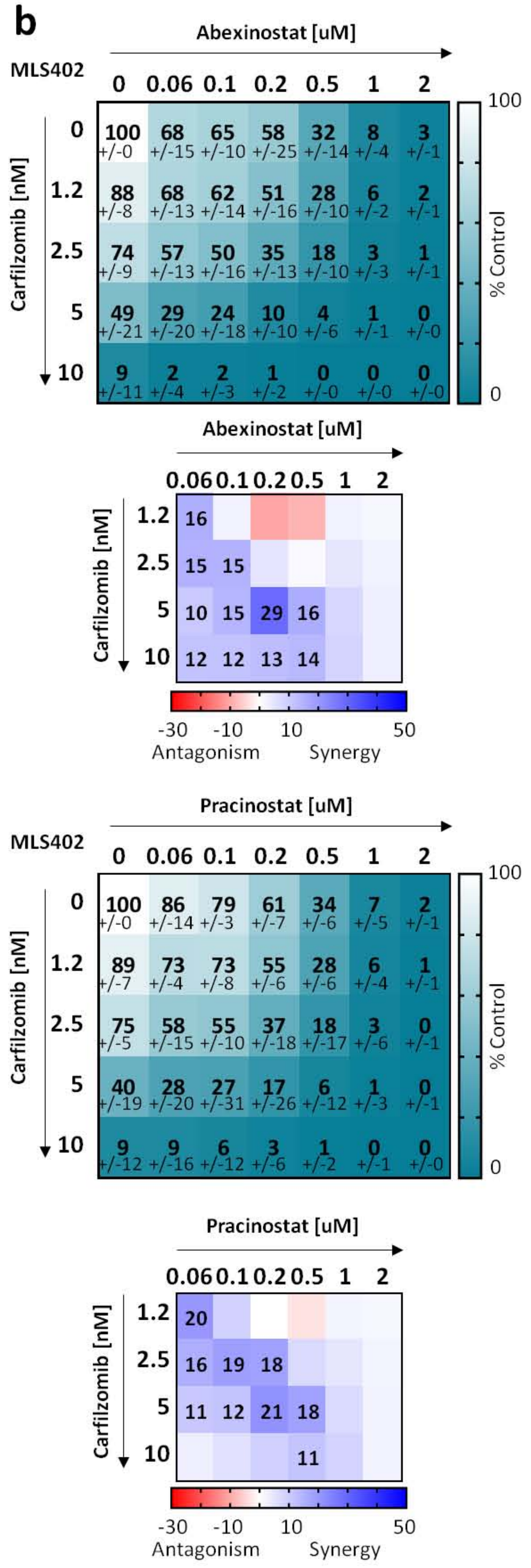

c

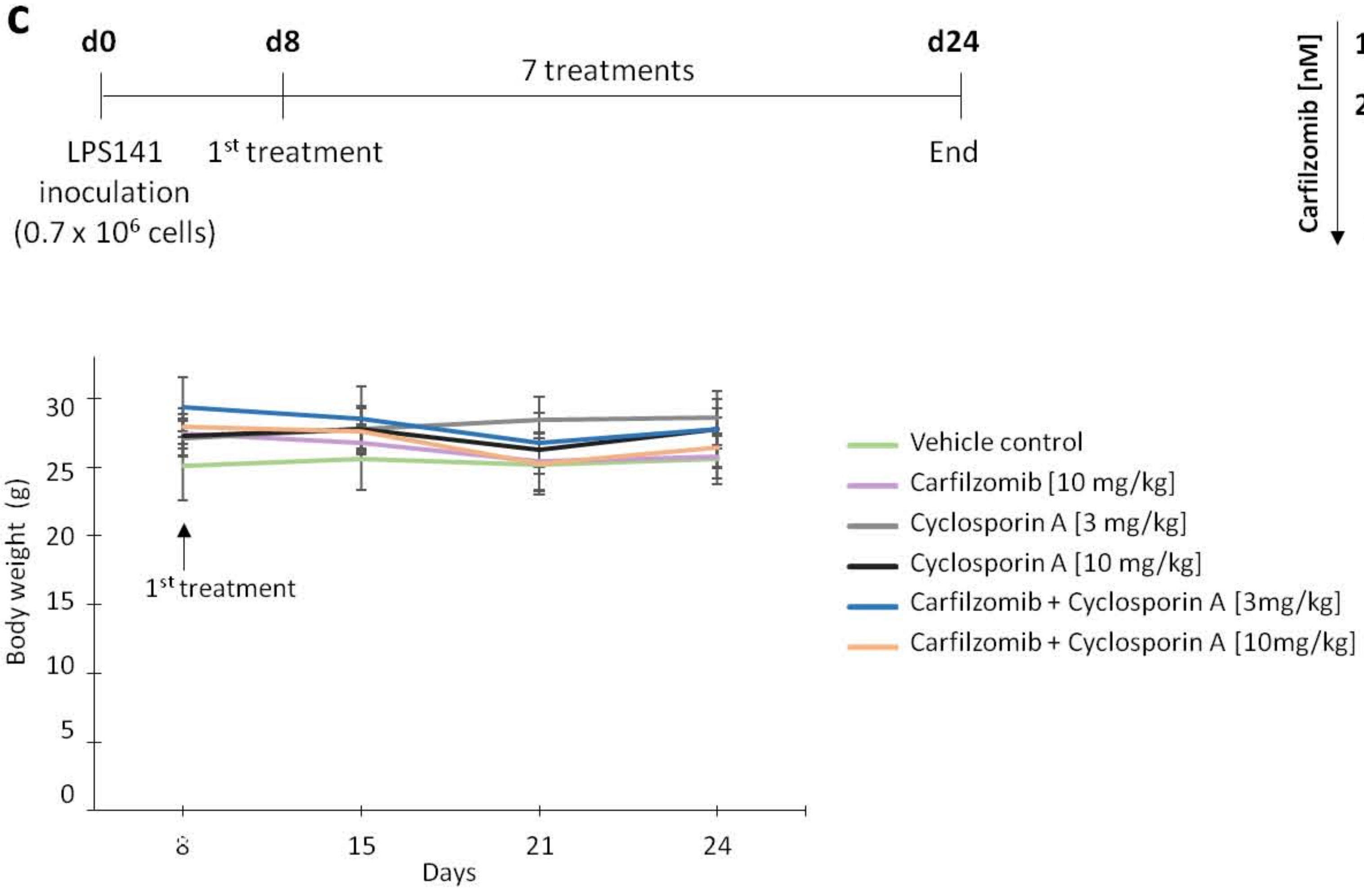

Supplement: Supplementary file 3 — Supplementary Figure S1: a) Viability of Adipose-derived Stem Cells (ASCs) after treatment with either carfilzomib or bortezomib. Values represent mean ± SD of at least triplicate. b) Representative clonogenic assay of LPS cells treated with Carfilzomib. c, d) Bodyweight graphs of mice used in Fig. 1e–g. Supplementary Figure S2: a) Viability assays of LPS141 cells treated with either carfilzomib, selinexor or combinations of both (n = 2 experiments performed in duplicate, values are mean ± SD). Right panel is a heatmap of the HSA synergy and antagonism scores.b) Colony formation assays of LPS141 cells treated with combinations of carfilzomib and selinexor. Left panel is a representative image of the assay. Middle panel represents relative absorbance values of each condition. Right panel depicts the HSA scores. c) Phospho-kinase arrays on MLS402 cells treated with either DMSO, carfilzomib (15 nM), selinexor (60 nM) or a combination of both. Antibodies against phosphorylated forms of 43 kinases and 2 related total proteins are spotted on the membrane in duplicate. A chemiluminescent signal represents phosphorylation of each protein. d) Quantification of signal intensities of the reference spots on the phospho-kinome array. Supplementary Figure S3: Two-dimensional SILAC ratio plots showing quantified proteins by mass spectrometry analysis in either LPS141 or MLS402 cells treated with either carfilzomib or bortezomib. Proteins in the top left quadrant of each plot are accumulated after proteasome inhibition, while those on the bottom right are down-regulated. Proteins annotated on the right side of each plot are those which accumulated (log2 fold change > 2) after treatment with either carfilzomib or bortezomib.Supplementary Figure S4: a-b) qRT-PCR showing relative expression of selected transcripts in either LPS141 non-treated (NT), treated with either DMSO, bortezomib (40 nM) or carfilzomib (80 nM) for 24 h. Values represent mean ± SEM of three experiments. c-d) Left [file 18_2020_3620_MOESM3_ESM.pdf]
