# Supplementary material for: Novel carfilzomib-based combinations as potential therapeutic strategies for liposarcomas
Source: Cell Mol Life Sci. 2020 Aug 26;78(4):1837–51. doi: 10.1007/s00018-020-03620-w (PMC7904719; doi:10.1007/s00018-020-03620-w)
Supplement: Supplementary file 4 — Supplementary material 4 (DOCX 25 kb) [file 18_2020_3620_MOESM4_ESM.docx]

**Supplementary Material and Methods**

**Source of cell lines**

LPS141, LP6 and FUDDLS1 cells (dedifferentiated LPS) were kindly provided by Dr. Nishio and Dr. Fletcher, respectively. MLS402 cells (Myxoid LPS) were kindly provided by Dr. Åman. T1000 cells (recurrent well-differentiated LPS) and LiSa-2 cells (metastatic poorly differentiated LPS) were kindly provided by Dr. Pedeutour and Dr. Moller, respectively. Human adipose-derived stem cells (ASCs) were purchased from LaCell LLC (New Orleans, LA, USA).

**Generation of shRNA cell lines**

ShRNA control vectors or targeting WNK1 or PRAS40 were engineered using the pLKO.1 lentiviral vector. Sequences used for shRNA vectors are the following (5’🡪 3’):

shControl:CCGGCAACAAGATGAAGAGCACCAACTCGAGTTGGTGCTCTTCATCTTGTTGTTTTTG

shWNK1:CCGGCCGCGATCTTAAATGTGACAACTCGAGTTGTCACATTTAAGATCGCGGTTTTTTG

shPRAS40:CCGGCCAGAAGCTGAAGCGGAAATACTCGAGTATTTCCGCTTCAGCTTCTGGTTTTTTG

LPS141 or MLS402 were transduced with fresh lentiviral particules and selected with puromycin for one week.

**MTT cell viability assays**

Around 1,500-2,000 cells were plated per well in 96-well plates. The following day, drugs were added at appropriate concentrations. Three days after treatment, MTT reagent (3-(4, 5-dimethylthiazolyl-2)-2, 5-diphenyltetrazolium bromide) was added to each well and plates were incubated at 37°C for 3-4 hours. Precipitates were solubilized by adding detergent reagent and incubating at room temperature in the dark. Absorbance was measured at 570 nm in a TecanInfinite^®^ 200 PRO microplate reader.

For shRNA studies, 2,000 cells of each cell line were plated on day 0 in 4 plates and one plate was stopped each day, for 4 days.

**Clonogenic assays**

Around 250 or 500 cells were seeded per well in 12-well or 6-well plates, respectively. The next day, drugs were added at desirable concentrations. Plates were incubated at 37°C for 10-15 days until visible colonies were detected. Colonies were then fixed with methanol and stained with crystal violet (0.5% w/v). Colonies were lysed with DMSO and corresponding absorbance was measured at 570nm in a Tecan Infinite^®^ 200 PRO microplate reader.

**Drugs**

Carfilzomib (HY-10455), bortezomib (HY-10227), KRIBB11 (HY-100872), cyclosporin A (HY-B0579), abexinostat (HY-10990) and pracinostat (HY-13322) were purchased from MedchemExpress. SC26196 (sc-361350) was purchased from Santa Cruz. Selinexor was a kind gift from Karyopharm Therapeutics. All drug stocks were prepared in DMSO and preserved at -80°C.

**SILAC labeling**

For SILAC labeling, LPS141 and MLS402 cells were incubated in RPMI 1640 (-Arg, -Lys) medium containing 10% dialyzed fetal bovine serum (PAN-Biotech) supplemented with 84 mg/l ^13^C_6_^15^N_4_ L-arginine and 50 mg/l ^13^C_6_^15^N_2_ L-lysine (Cambridge Isotope) or the corresponding non-labeled amino acids, respectively. Successful SILAC incorporation was verified by in-gel trypsin digestion and MS analysis of ‘heavy’ input samples to ensure an incorporation rate of > 98%.

**Mass spectrometry analysis**

Equal amounts of corresponding heavy or light-labeled DMSO or drug-treated extracts were mixed and 200 µg of protein were used for MS sample preparation. Heavy and light mixed samples were boiled at 95°C prior to separation on a 4-12% NuPAGE Bis-Tris precast gel (Thermo Fisher Scientific) for 60 min at 170 V. The gel was fixed using the Colloidal Blue Staining Kit (Thermo Fisher Scientific) and each lane was divided into 8 equal fractions. For in-gel digestion, samples were destained (25 mM ammonium bicarbonate; 50% ethanol), reduced in 10 mM DTT for 1h at 56°C followed by alkylation with 55mM iodoacetamide (Sigma) for 45 min in the dark. Tryptic digest was performed with 2 μg trypsin (Promega) in 50 mM ammonium bicarbonate buffer at 37°C overnight. Peptides were desalted on StageTips and analysed by nanoflow liquid chromatography on an EASY-nLC 1200 system coupled to a Q Exactive HF mass spectrometer (Thermo Fisher Scientific). Peptides were separated on a C18-reversed phase column (25 cm long, 75 μm inner diameter) packed in-house with ReproSil-Pur C18-AQ 1.9 μm resin (Dr Maisch). The column was mounted on an Easy Flex Nano Source and temperature controlled by a column oven (Sonation) at 40°C. A 215-min gradient from 2 to 40% acetonitrile in 0.5% formic acid at a flow of 225 nl/min was used. Spray voltage was set to 2.4 kV. The Q Exactive HF was operated with a TOP20 MS/MS spectra acquisition method per MS full scan. MS scans were conducted with 60,000 at a maximum injection time of 20 ms and MS/MS scans with 15,000 resolution at a maximum injection time of 50 ms. The raw files were processed with MaxQuant [1] version 1.5.2.8 with preset standard settings for SILAC labeled samples and the re-quantify option was activated. Carbamidomethylation was set as fixed modification while methionine oxidation and protein N-acetylation were considered as variable modifications. Search results were filtered with a false discovery rate of 0.01. Known contaminants, proteins groups only identified by site, and reverse hits of the MaxQuant results were removed and only proteins were kept that were quantified by SILAC ratios in both ‘forward’ and ‘reverse’ samples. The mass spectrometry proteomics data have been deposited to the ProteomeXchange Consortium via PRIDE [2] repository with the dataset identifiers PXD021001 and PXD020906.

**Western blot and SDS-PAGE electrophoresis**

Cell pellets were lysed in RIPA buffer, and protein concentrations estimated using BCA reagent (Santa Cruz). Protein loading buffer was added and samples were incubated for 10 min at 95°C. Samples were loaded in 10% gels and SDS-PAGE electrophoresis performed at 160V for 1 hour. Samples were transferred to PVDF membrane (Merck Millipore) at either 70V for 3 hours or 40V overnight. After transfer, the membrane was blocked in TBS, 5% Milk, 0.1% Tween 20 for 1 hr and then incubated with the primary antibody diluted in TBS, 3% Milk, for 1hr at RT or overnight at 4°C. The membrane was incubated with secondary antibodies coupled to HRP diluted in TBS, 3% Milk. Washes after each antibody incubation were performed with TBS, 0.1% Tween 20. Protein bands were visualized by adding SuperSignal West Femto Chemiluminescent Substrate (Thermo Fisher Scientific).

**List of antibodies**

Ubiquitin (Cell Signaling, #3933); CCNB1 (Santa Cruz Biotechnology, SC-245); AURKA (Santa Cruz Biotechnology, SC-398814); CENPE (Santa Cruz Biotechnology, SC-376685); FADS2 (GeneTex, GTX64748); HERC4 (Novus Biologicals, NBPI-00104); BAG3 (Santa Cruz Biotechnology, SC-136467); HMOX1 (Santa Cruz Biotechnology, SC-398814); GAPDH (Cell Signaling, #2118); β-actin (Sigma-Aldrich, A5441). All antibodies were used at a dilution of 1:1000.

**Quantitative real-time PCR**

Total RNA was isolated using ReliaPrep RNA Cell Miniprep system kit (Promega), and cDNA synthesized using EvoScript Universal cDNA Master kit (Roche). For Quantitative real-time PCR, diluted cDNA was mixed with desired primers and Precision Fast qPCR MasterMix - Low Rox- SYBR, and reactions performed in 7500 Fast Real-time cycler (Applied Biosystems).

Gene specific primers (5’ 🡪 3’) were designed using PrimerBank database [3]:

FADS2_Forward: TGACCGCAAGGTTTACAACAT

FADS2_Reverse: AGGCATCCGTTGCATCTTCTC

TPX2_Forward: ACTTCCGCACAGATGAGCG

TPX2_Reverse: GGATGCTTTCGTAGTTCAGATGT

SESN2_Forward: TCTTACCTGGTAGGCTCCCAC

SESN2_Reverse: AGCAACTTGTTGATCTCGCTG

UBES_Forward: CCGACACGTACTGCTGACC

UBES_Reverse: GCCGCATACTCCTCGTAGTTC

NUSAP1_Forward: AGCCCATCAATAAGGGAGGG

NUSAP1_Reverse: ACCTGACACCCGTTTTAGCTG

CCNB1_Forward: AATAAGGCGAAGATCAACATGGC

CCNB1_Reverse: TTTGTTACCAATGTCCCCAAGAG

HSPA6_Forward: CATCGCCTATGGGCTGGAC

HSPA6_Reverse: GGAGAGAACCGACACATCGAA

BAG3_Forward: TGGGAGATCAAGATCGACCC

BAG3_Reverse: GGGCCATTGGCAGAGGATG

HMOX1_Forward: AAGACTGCGTTCCTGCTCAAC

HMOX1_Reverse: AAAGCCCTACAGCAACTGTCG

IRFD1_Forward: GAGTGCGAAGACAAGGCAAG

IRFD1_Reverse: GCAGCGTTCAATGCTATCAGTT

WNK1_Forward: GCCGTCAGATCCTTAAAGGTC

WNK1_Reverse: CCAGTAGGGCCGGTGATAA

PRAS40_Forward: CTACCCACACAGCAGTACGC

PRAS40_Reverse: AAGTCGCTGGTGTTAAGCCG

GAPDH_Forward: GTCGCCAGCCGAGCCACATC

GAPDH_Reverse: GGTGACCAGGCGCCCAATACG

**Supplementary references**

1. Cox, J. and M. Mann, *MaxQuant enables high peptide identification rates, individualized p.p.b.-range mass accuracies and proteome-wide protein quantification.* Nat Biotechnol, 2008. **26**(12): p. 1367-72.

2. Vizcaino, J.A., et al., *2016 update of the PRIDE database and its related tools.* Nucleic Acids Res, 2016. **44**(22): p. 11033.

3. Wang, X., et al., *PrimerBank: a PCR primer database for quantitative gene expression analysis, 2012 update.* Nucleic Acids Res, 2012. **40**(Database issue): p. D1144-9.
